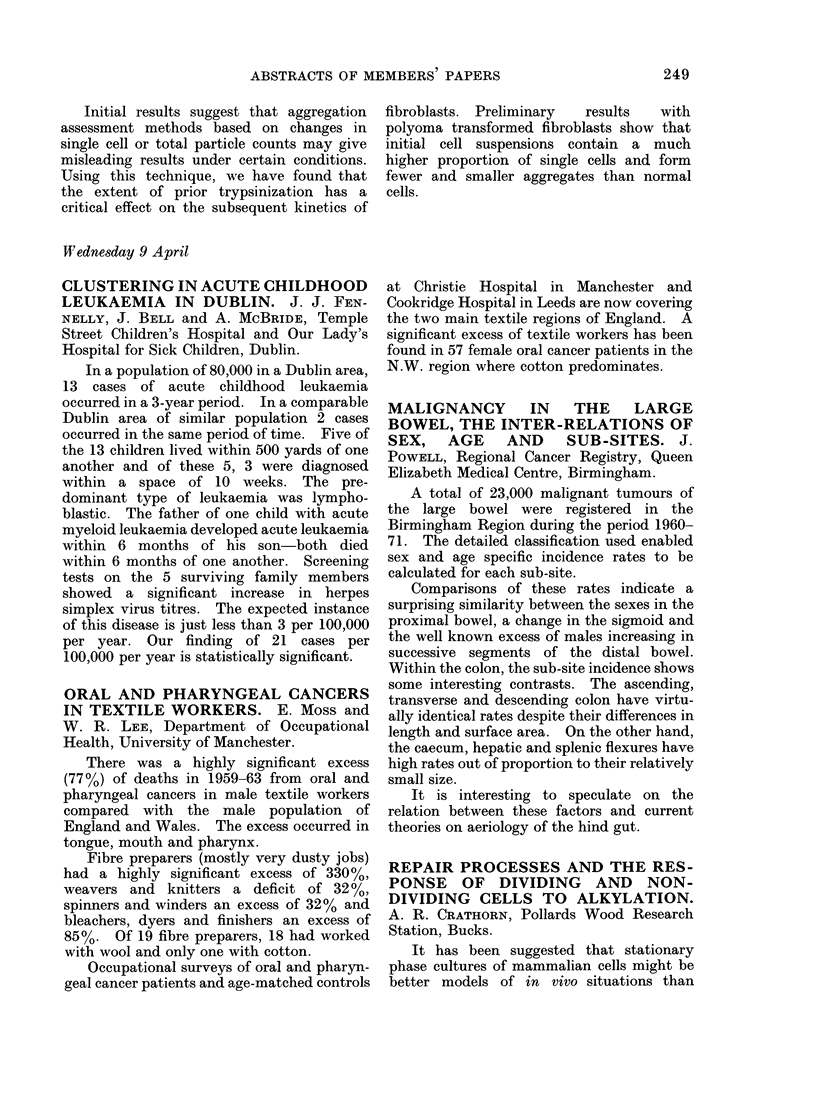# Proceedings: Oral and pharyngeal cancers in textile workers.

**DOI:** 10.1038/bjc.1975.185

**Published:** 1975-08

**Authors:** E. Moss, W. R. Lee


					
ORAL AND PHARYNGEAL CANCERS
IN TEXTILE WORKERS. E. Moss and
W. R. LEE, Department of Occupational
Health, University of Manchester.

There was a highly significant excess
(77%) of deaths in 1959-63 from oral and
pharyngeal cancers in male textile workers
compared with the male population of
England and Wales. The excess occurred in
tongue, mouth and pharynx.

Fibre preparers (mostly very dusty jobs)
had a highly significant excess of 330%,
weavers and knitters a deficit of 32%,
spinners and winders an excess of 32% and
bleachers, dyers and finishers an excess of
85%. Of 19 fibre preparers, 18 had worked
with wool and only one with cotton.

Occupational surveys of oral and pharyn-
geal cancer patients and age-matched controls

fibroblasts. Preliminary  results   with
polyoma transformed fibroblasts show that
initial cell suspensions contain a much
higher proportion of single cells and form
fewer and smaller aggregates than normal
cells.

at Christie Hospital in Manchester and
Cookridge Hospital in Leeds are now covering
the two main textile regions of England. A
significant excess of textile workers has been
found in 57 female oral cancer patients in the
N.W. region where cotton predominates.